# Inhibition of Phosphodiesterase-4 Reverses Aβ-Induced Memory Impairment by Regulation of HPA Axis Related cAMP Signaling

**DOI:** 10.3389/fnagi.2018.00204

**Published:** 2018-07-24

**Authors:** Ying Xu, Naping Zhu, Wen Xu, Han Ye, Kaiping Liu, Feiyan Wu, Meixi Zhang, Yun Ding, Chong Zhang, Hanting Zhang, James O'Donnell, Jiangchun Pan

**Affiliations:** ^1^Brain Institute, School of Pharmacy, Wenzhou Medical University, Wenzhou, China; ^2^Department of Pharmaceutical Sciences, School of Pharmacy and Pharmaceutical Sciences, University at Buffalo, State University of New York, Buffalo, NY, United States; ^3^Pingyang Hospital of Traditional Chinese Medicine, Pingyang, China; ^4^Hangzhou Geriatric Hospital, Hangzhou, China; ^5^Departments of Behavioral Medicine, Psychiatry and Physiology, and Pharmacology, West Virginia University Health Sciences Center, Morgantown, WV, United States

**Keywords:** Alzheimer's disease, Aβ1-42, rolipram, learning and memory, Phosphodiesterases 4A, HPA axis

## Abstract

Beta amyloid peptides (Aβ) are found to be associated with dysfunction of hypothalamic-pituitary-adrenal axis (HPA axis) that leads to memory and cognitive deficits in patients with Alzheimer's disease (AD). Phosphodiesterase 4 (PDE4) inhibitors increase the intracellular cAMP activities, which may ameliorate cognitive deficits associated with AD. However, it remains unclear whether PDE4-mediated reversal of cognitive impairment in mouse model of AD is related to HPA axis and downstream cAMP-dependent pathway. The present study investigated the effects of PDE4 inhibitor rolipram on Aβ1-42-induced cognitive dysfunction and its underlying mechanisms. The step-down passive avoidance (PA) and Morris water-maze (MWM) tests were conducted 1 week (1 W), 2 months (2 M), and 6 months (6 M) after intracerebroventricular microjection (i.c.v.) of Aβ1-42. The results suggested that memory impairment emerged as early as 1 W, peaked at 2 M, and lasted until 6 M after injection. Chronic treatment with rolipram (0.1, 0.5, 1.0 mg/kg/d, i.p.) for 2 weeks (i.e., treatment started at 1.5 months after Aβ1-42 microinjection) dose-dependently improved memory performance in both MWM and PA tests. Moreover, rolipram reversed the Aβ-induced increases in serum corticosterone (CORT), corticotropin-releasing factor, and glucocorticoid receptors (CRF-R and GR) levels, whereas it decreases in brain-derived neurotropic factor (BDNF) and the ratio of pCREB to CREB expression. These effects of rolipram were prevented by pre-treatment with PKA inhibitor H89. The findings indicated that the protective effects of rolipram against Aβ1-42-induced memory deficits might involve HPA axis and cAMP-CREB-BDNF signaling.

## Introduction

Alzheimer's disease (AD), the most common type of dementia, is a progressive nervous system degenerative disease characterized by subjective cognitive decline (SCD) and mild cognitive impairment (MCI) (Heckman et al., 2015). The amyloid cascade hypothesis has been considered as the central hypothesis for the cause of AD, which suggests that memory deficits correlate closely with cortical soluble beta amyloid (Aβ) oligomers and protofibrils (Blennow et al., 2006). Two primary forms of Aβ, such as Aβ1-42 and Aβ1-40, are recognized as disease markers in AD patients. However, Aβ1-42 form is more neurotoxic than the Aβ1-40 form, which mainly facilitates the progression of the disease (Cheng et al., 2010). Recent studies suggested that it is not sufficient to decrease the cerebral Aβ levels for the purpose of preventing memory impairment because the accumulation of beta amyloid or senile plaques is often comorbid with increased neurofibrillary tangles (Ballard et al., 2011), making the symptoms irreversible in the later period of the disease. Therefore, early intervention with medicine is necessary to counteract the progress of memory deficits and dementia.

The hypothalamic-pituitary-adrenal (HPA) axis is an intricate arrangement of direct impacts and feedback interactions among three endocrine glands, i.e., the hypothalamus, pituitary, and adrenal glands. Increasing evidence suggests that animal models with MCI experience moderately increased corticosterone levels in serum, which indicate that dysfunction of the HPA axis may lead to memory decline that occurs in early dementia (Xu et al., 2009). Indeed, clinical studies have suggested that cognitive dysfunctions (i.e., learning and memory deficits) and psychological symptoms (i.e., irritation, depression, or anxiety) in AD patients are related to an early disturbance of the HPA axis (Brureau et al., 2013). However, whether such impacts involve Aβ accumulation is still controversial (Green et al., 2006). Our previous studies suggested that dysregulation of HPA axis decreased the second messenger cyclic nucleotides, 3′5′-cyclic guanosine monophosphate (cGMP) and 3′5′-cyclic adenosine monophosphate (cAMP) (Xu et al., 2013, 2015), which are important learning and memory mediators in various species of animals (Lueptow et al., 2016). Therefore, targeting the cyclic nucleotide phosphodiesterases (PDEs), the enzymes that hydrolyze and inactivate these second messengers, is expected to enhance cognition. PDE4, one of the PDE enzymes that selectively hydrolyzes cAMP, is considered to be involved in learning and memory processes (Richter et al., 2013). The selective PDE4 inhibitor has a series of CNS effects including antidepressant and memory-enhancing effects (Richter et al., 2013). Recent study suggested that PDE4 inhibitor could ameliorate the HPA axis dysfunction induced by stress (Jindal et al., 2015), but studies linking dysfunction of HPA axis caused by Aβ and treatment of PDE4 inhibitors are lacking.

In the present study, the serum corticosterone level, glucocorticoid receptor (GR) and corticotropin-releasing factor receptor (CRF-R) expression, phosphorylation of cyclic AMP response element binding (pCREB), and brain-derived neurotrophic factor (BDNF) levels were examined to clarify whether Aβ1-42-induced pathological changes were related to HPA axis dysfunction, and how PDE4 inhibition could enhance memory through the regulation of HPA axis system.

## Materials and methods

### Animals

Adult male ICR mice (22–25 g) used for the experiments were obtained from the Animal Center of Shanghai Branch, Chinese Academy of Sciences. Mice were housed in a temperature-controlled room with the humidity of 50–60% and a 12-h light/dark cycle and had free access to standard diet and water. Mice were allowed an acclimation period for 5 days before the experiments. All procedures were carried out in a quiet room according to the “NIH Guide for the Care and Use of Laboratory Animals” (NIH Publications No. 80-23, revised 1996) and were approved by Committees of Wenzhou Medical University on Animal Care and Use.

### Surgery

The surgery for brain cannula implantation was performed aseptically under xylazine (6 mg/kg) and ketamine (100 mg/kg) anesthesia. The mice were placed in a stereotaxic apparatus with flat-skull position and the head was kept horizontally. The guide cannulas (30-gauge) were implanted bilaterally into the intracerebroventricles (AP −0.2 mm from bregma, ML ± 1.0 mm from midline, DV −2.5 mm from dura) (Wang et al., 2016) for microinfusion. Three days after the surgery, 2 μl Aβ1-42 (0.4 μg/μl, 1 μl/side) or 0.9% sterile saline was infused bilaterally at the rate of 0.25 μl/min using microinjection pump. To permit diffusion after microinjection, the infusion cannulae were left in place for an extra 5 min.

### Chemicals and drug administration

Aβ1-42 (rPeptide, USA) was dissolved in 0.9 % sterile saline, at a final concentration of 0.4 μg/μl, and incubated at 37°C for 4 days to obtain aggregated Aβ before microinfusion into cerebroventricle (Wang et al., 2012). Rolipram (Sigma-Aldrich, USA) was prepared by being dissolved in 0.9% sterile saline containing 1% dimethyl sulfoxide (DMSO). One and a half months after microinjection with Aβ1-42, the mice were treated with different doses of rolipram (0.1, 0.5, 1.0 mg/kg/day, i.p.) or vehicle for 2 weeks. KT5823 (Cayman Chemical, USA), a selective inhibitor of cGMP-dependent protein kinase (PKG), and H89 (Sigm-Aldrich, USA), a cAMP-dependent protein kinase (PKA), were dissolved in artificial cerebrospinal fluid (ACSF) and were bilaterally microinjected into the intracerebroventricular, 30 min before treatment with rolipram.

The primary antibodies of anti-CRFR1, anti-BDNF, and anti-GR were purchased from Abcam Biotechnology Company (Abcam, Cambridge, MA). The anti-pCREB and anti-CREB were purchased from Merck Milipore (Millipore,Billerica,MA,USA). All the secondary antibodies (anti-rabbit lgG) were purchased from MultiSciences Biotech Co., Ltd. (MultiSciences, Hangzhou, China). The CORT ELISA kit was purchased from Enzo Life Sciences (Enzo Life Sciences, USA).

### Behavioral test procedures

Two experiments were included in this project. In experiment 1, six groups of 10 mice each were used for measuring the learning and memory performances in the Morris water maze and step-down passive avoidance tests (MWM and PA). The six groups are (1) control (1 W), (2) model (1 W), (3) control (2 M), (4) model (2 M), (5) control (6 M), and (6) model (6 M). Experiment 2 was performed to investigate the effects of chronic treatment of rolipram on short-term and long-term memory processes using the MWM and PA tests. Specifically, seven groups of 10 mice each were used for this experiment: (1) vehicle 1 (for Aβ1-42) + vehicle 2 (for rolipram), (2) Aβ1-42 + vehicle 2, (3) Aβ1-42 + rolipram (0.1 mg/kg, i.p.), (4) Aβ1-42 + rolipram (0.5 mg/kg, i.p.), (5) Aβ1-42 + rolipram (1.0 mg/kg, i.p.), (6) Aβ1-42 + rolipram (1.0 mg/kg, i.p.) + KT5823 (20 μM, 1 μl/side), and (7) Aβ1-42 + rolipram (1.0 mg/kg) + H89 (5 μM, 1 μl/side). They were injected drugs once daily for 2 weeks before MWM and PA tests, i.e., 1.5 months after i.c.v. injections of Aβ1-42. H89 (5 μM, 1 μl/side) and KT 5823 (20 μM, 1 μl/side) were given 30 min before treatment with rolipram. H89 and KT5823 used alone did not show any effects, which were summarized in Supplementary Figures 1, 2.

### Morris water maze test (MWM)

The MWM test was conducted as described earlier (Xu et al., 2015). The circular pool was filled with opaque water (21 ± 1°C). A hidden circular platform (8.5 cm diameter and 15.5 cm high) was submerged 2 cm under the surface of water in the fourth quadrant. The acquisition trials were carried out for six learning blocks before the probe trial and separated by 20 min intervals. Each block includes 3 trials, during which the mice were placed in the apparatus from the first, second, or third quadrant. Mice were guided to the platform manually unless they escaped to the hidden platform. During the acquisition trials, the platform was invisible in the similar area relative to the distal cues in the room. The probe trial was conducted 1 and 24 h after the training session to measure the spatial memory, in which the platform was removed. During the probe trial, mice were placed in the apparatus from the second quadrant, which was farthest from the hidden platform located before. The number of platform crossings and the latency to reach the previous platform location were recorded for comparison.

### Step-down passive avoidance test (PA)

The test was carried out utilizing a square chamber within a wooden platform on one side of the grid floor, which can receive electric shocks from the isolated pulse stimulator. The test consists of 3 sessions: habituation, training, and retention. Preceding the training, mice were first habituated to the apparatus. During the habituation process, the mice were separately placed on the platform. Once their feet were fully exposed to the grid floor, mice were subjected to a foot shock (0.4–0.8 mA, 40 V, 0.5 s, 50 Hz, 20 s intertribal interval). The training test repeated the habituation procedure after 1 h. Mice were considered to have taken in the task when they stayed on the platform for over 60 s. Retention tests were conducted 3 and 24 h after the training session. During the retention session, the electric shock of the grid was removed. The mouse was individually placed on the wooden platform and the time of first attempt to jump off the platform was recorded as step-down latency, with an upper cut off time of 300 s (Nasehi et al., 2012).

### Serum corticosterone measurement

The serum level of CORT was assessed by ELISA assay based on the manufacturer's instructions. Following behavioral testing, the mice were decapitated and the trunk blood was collected, the plasma was removed by centrifuging at 3,000 rpm for 10 min (Xu et al., 2013). In order to rule out the potential effects of diurnal rhythm on mouse hormone levels, all blood samples were collected at 4:00–6:00 pm.

### Immunoblotting analysis

Mice were decapitated following the behavioral tests. The brain tissues including the hippocampus and prefrontal cortex were dissected and immediately stored at −80°C until analysis. They were then thawed and subsequently homogenized in RIPA lysis buffer containing protease and phosphatase inhibitors and centrifuged at 14,000 rpm for 20 min at 4°C for the measurement of protein. Protein concentrations were determined using the BCA protein assay (Chen et al., 2011). Samples (60 μg protein each) were separated using SDS-PAGE before transferring to PVDF membranes (0.20 μm; Millipore, Billerica, MA, USA). Nonspecific bindings were blocked with 5% skim milk for 90 min. Membranes were subsequently incubated with the appropriate primary antibodies for rabbit anti-GR (1:5,000), anti-CRF-R (1:5,000), anti-pCREB (1:1,000), anti-CREB (1:500), anti-BDNF (1:2,000), and anti-β-Actin (1:1,000) overnight at 4°C. After washing with TBST (0.1%) and incubation with secondary antibodies, ECL kit was used to visualize the immune complex by chemiluminescence. The specific bands were detected using GelDoc XR System (Bio-Rad, USA) and quantified using Quantity One software.

### Statistical analysis

Data shown are expressed as means ± S.E.M. For multiple comparisons, data were analyzed using one-way analysis of variance (ANOVA) followed by a post hoc Dunnett's test. For two group's comparisons, data were analyzed statistically using Student's *t*-test. A *p* < 0.05 was considered to be significant.

## Results

### The effects of Aβ1-42 on spatial learning and memory in the MWM at 1 W, 2 M, and 6 M

**Figure 1:**
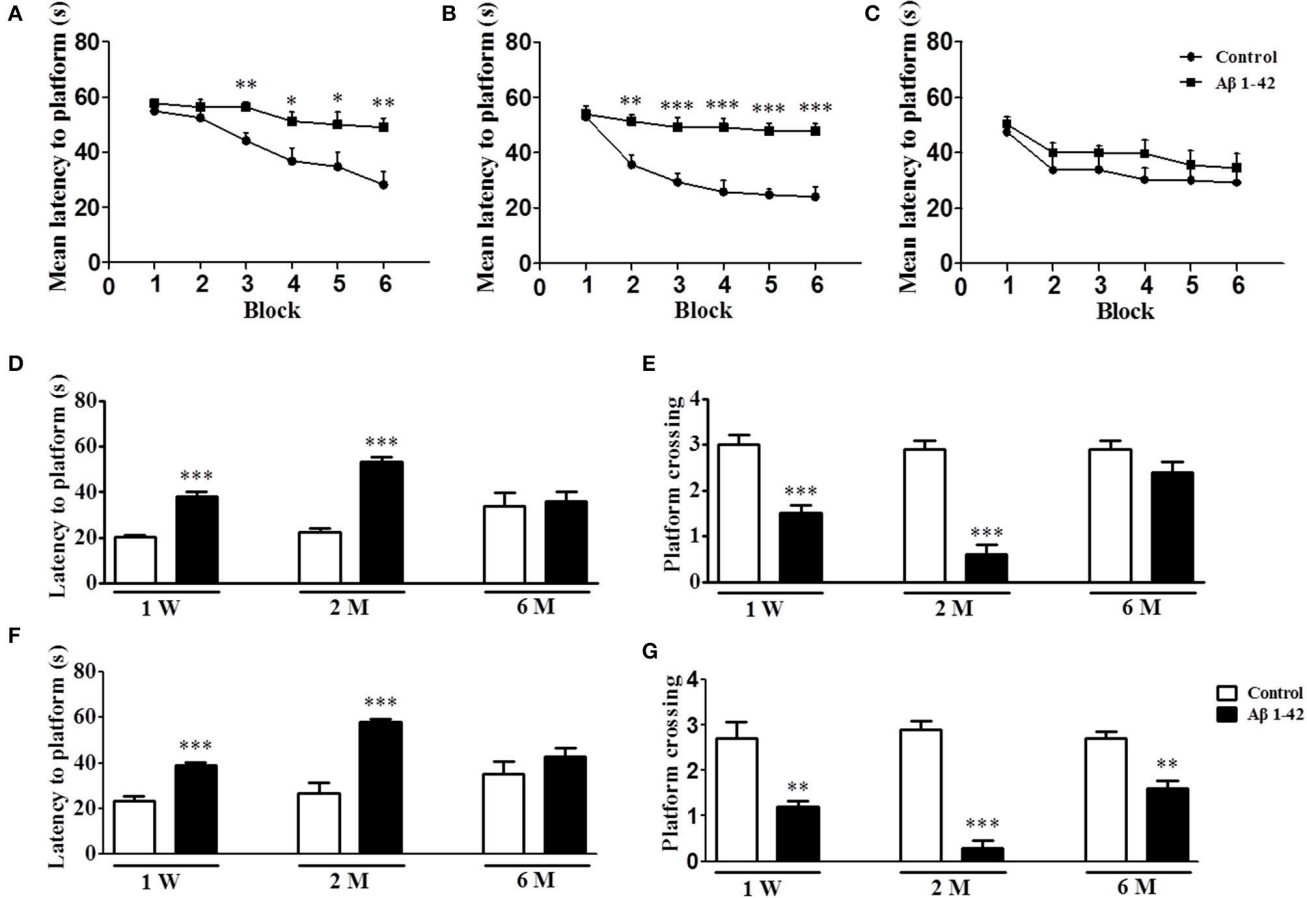
Aβ1-42 induced spatial memory impairment in the Morris water maze task at 1 week, 2 and 6 months after intracerebroventricular microinjection of Aβ1-42. During the acquisition trials of the water maze task, learning curve were tested after intracerebroventricular microinjection of Aβ1-42 1 week **(A)**, 2 months **(B)**, and 6 months **(C)**. The probe trial was conducted 1 and 24 h after training, the latency to reach the platform **(D,F)** and the number of platform crossings **(E,G)** were determined. Values shown are means ± SEM, *n* = 10; ^*^p < 0.05, ^**^p < 0.01, and ^***^p < 0.001 vs. vehicle-treated control group.

As shown in Figure 1, intracerebroventricular injection of Aβ1-42 altered the learning ability of mice in the MWM as compared with control animals at 1 W, 2 M, and 6 M. There were no significant differences in mean latency time in the initial training block at any time point (Figure 1A–C). Animals treated with Aβ1-42 at 1 W prior to the test exhibited partial reduction in learning in MWM, as was demonstrated by significantly increased mean latency to platform from block 3 to block 6 (*p* < 0.01, *p* < 0.05, *p* < 0.05, *p* < 0.01, Figure 1A). The most prominent learning impairment was found at 2 M, in which the latency to platform was significantly longer from block 2 to block 6 (*p* < 0.01, *p's* < 0.001, Figure 1B). At 6 M, the Aβ-treated animals did not exhibit significantly increased latency to platform as compared with those of controls. Swimming speed did not change (data not shown).

Figures 1D,E presented the data on short-term memory. At each time point, i.e., 1 W, 2 M, and 6 M, 1 h after training, the hidden platform was removed and the probe trial was conducted. The results showed that Aβ1-42-treated mice exhibited increased latencies to platform at 1 W and 2 M (*p's* < 0.001, Figure 1D). At the same time points, platform crossings were also significantly reduced in the Aβ1-42-treated mice as compared with the vehicle-treated mice (*p's* < 0.001, Figure 1E). The results of Figures 1F,G were from the 24 h probe trial, which was used for examining the long-term memory. The data showed that Aβ1-42-treated animals exhibited increased latencies to platform at 1 W and 2 M (*p's* < 0.001, Figure 1F); and at all the time points, i.e., 1 W, 2 M and 6 M, Aβ1-42-treated mice exhibited reduced number of platform crossings as compared with the vehicle-treated animals (*p's* < 0.001, Figure 1G). Swimming speed also did not change (data not shown).

### The effects of Aβ1-42 on memory retention in the step-down passive avoidance test at 1 W, 2 M, and 6 M

**Figure 2:**
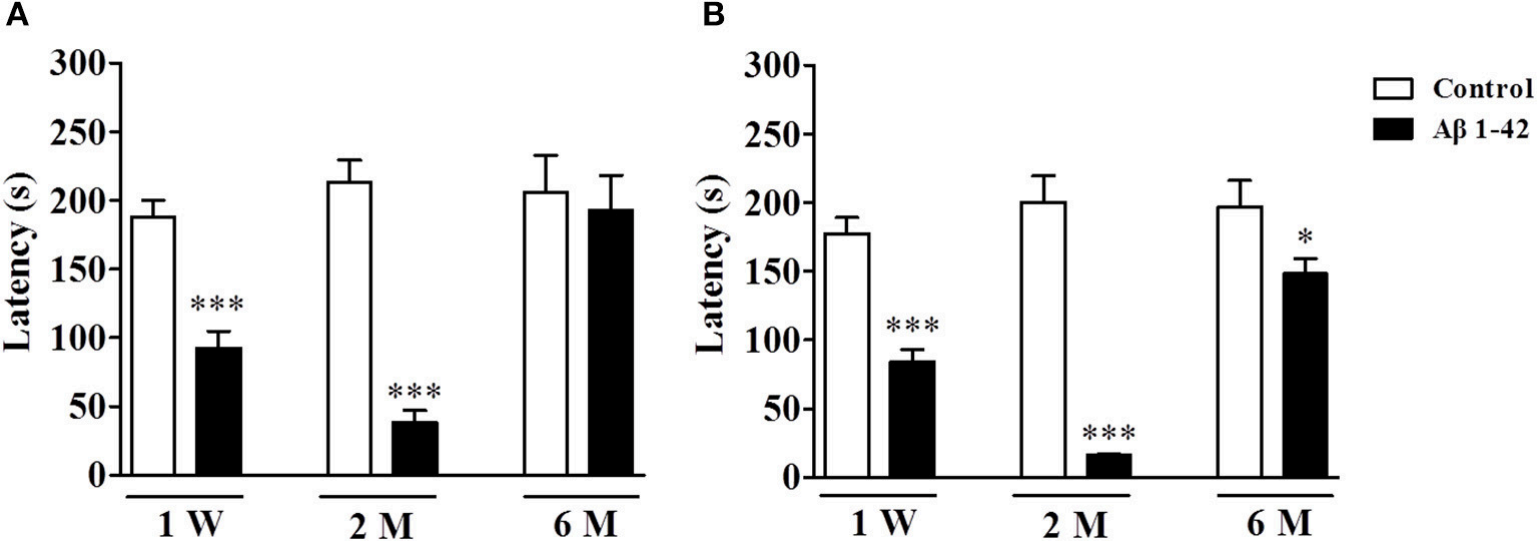
Aβ1-42 induced memory impairment in the step-down passive avoidance test at 1 week, 2 and 6 months after intracerebroventricular microinjection of Aβ1-42. Retention tests were conducted 3 **(A)** and 24 h **(B)** after the training session. Values shown are means ± SEM, *n* = 10; ^*^p < 0.05 and ^***^p < 0.001 *vs*. vehicle-treated control group.

The memory retention measured by the latency to jump off the platform was determined 3 and 24 h after the training session in the step-down passive avoidance test after the mice were treated with Aβ1-42 at different time periods, i.e., 1 W, 2 M, and 6 M, as shown in Figure 2. The results suggested that the short-term memory retention (3 h after training session) was significantly decreased after the treatment with Aβ1-42 at 1 W and 2 M when compared with vehicle-treated control groups (*p's* < 0.001, Figure 2A), which peaked at 2 M. However, the change was not significant 6 M after Aβ1-42 treatment. For long-term memory (24 h after training session), the differences of latency to the previous platform location were kept significant at 1 W, 2 M, and 6 M after Aβ1-42 treatment as compared with vehicle-treated control groups (*p* < 0.001; *p* < 0.05; Figure 2B).

### Rolipram reversed the Aβ-induced memory impairment in the MWM and PA tests

**Figure 3:**
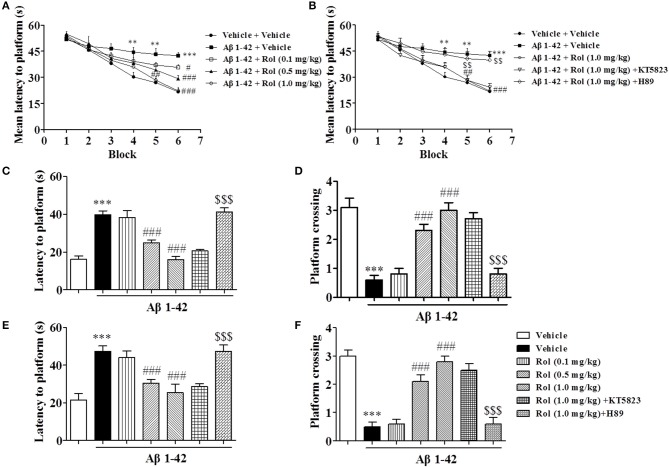
The effects of rolipram on Aβ1-42-induced memory impairment in the Morris water-maze test in mice. One and a half months after microinfusion with Aβ1-42 into cerebroventricle, mice were administrated with rolipram for 14 days. H89 and KT5823 were pretreated 30 min before rolipram administration every day. During the acquisition trials of the water maze task, learning curve was tested 24 h after last treatment with rolipram **(A,B)**. The probe trial was conducted 1 and 24 h after training, the latency to reach the platform **(C,E)** and the number of platform crossings **(D,F)** were determined. Values shown are means ± SEM, *n* = 10; ^**^p < 0.01 and ^***^p < 0.001 *vs*. vehicle treated sham group. ^#^*p* < 0.05, ^##^*p* < 0.01, and ^###^*p* < 0.001 *vs*. vehicle-treated Aβ group. ^$$^*P* < 0.01 and ^$$$^*P* < 0.001 *vs*. rolipram (1.0 mg/kg)-treated Aβ group.

Since serious memory impairment was found at 2 months after treatment with Aβ1-42, the following behavioral and biological assays were performed at this time point. During 6 blocks of training sessions in the MWM, the Aβ-treated mice presented an increase in mean latency to reach the platform from block 4 to 6 as compared with the vehicle-treated control group (*p's* < 0.01, *p* < 0.001, Figure 3A). Chronic rolipram administration (0.1, 0.5, and 1.0 mg/kg for 14 days) significantly reduced the mean latency to the platform from block 5 to 6 in Aβ1-42-treated mice, in a dose-dependent manner [*F*_(3, 36)_ = 30.22, *p* < 0.001]. However, these effects of rolipram on acquisition of memory, shown as the learning curve, were inhibited by the PKA inhibitor H89, which was administered 30 min before rolipram (*p's* < 0.01; Figure 3B). Meanwhile, Pretreatment with the PKG inhibitor KT5823 did not show such effects. Swimming speed did not change (data not shown).

The probe trial test was conducted 1 h after the last training session to assess the short-term spatial memory. The results suggested that Aβ1-42, administered 2 months prior to the MWM test, increased the latency to the previous platform location and decreased the number of platform crossings as compared with the vehicle-treated controls (*p's* < 0.001, Figures 3C,D). These effects were reversed by rolipram at doses of 0.5 and 1.0 mg/kg (i.p.) (*p's* < 0.001). Long-term spatial memory was also determined in the probe trial 24 h later. Memory performance was worse in the Aβ-treated mice, as indicated by an increased latency time to reach the previous platform location (*p* < 0.001, Figure 3E) and fewer platform crossings (*p* < 0.001, Figure 3F). Rolipram (0.1, 0.5, and 1.0 mg/kg, i.p.) ameliorated the effect of Aβ1-42 on latency to platform and platform crossing in a dose-dependent manner [*F*_(3, 36)_ = 9.739, *p* < 0.001, Figure 3E; *F*_(3, 36)_ = 34.567, *p* < 0.001, Figure 3F]. However, the amelioration due to high doses of rolipram (1.0 mg/kg) on both short-term and long-term memories was blocked by the pretreatment with H89 (*p's* < 0.001), but not KT5823. Swimming speed also did not change (data not shown).

**Figure 4:**
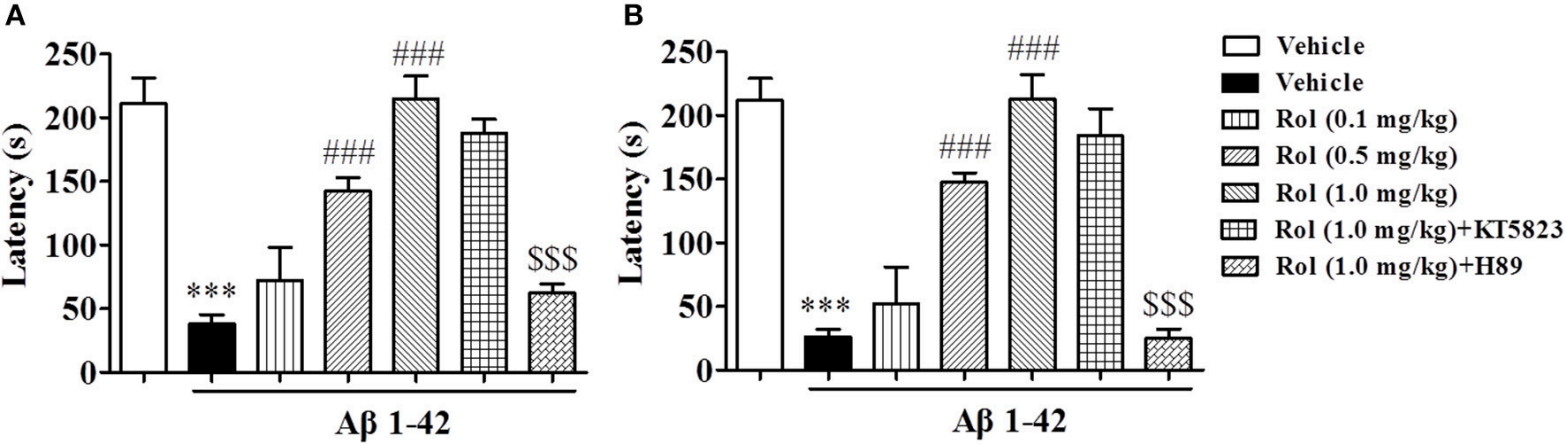
The effects of rolipram on 3-h **(A)** and 24-h **(B)** memory retention in the step-down passive avoidance test in Aβ42-treated mice. Aβ42-induced decreases in 3-h and 24-h retention were reversed by chronic treatment with rolipram for 14 days Values shown are means ± SEM, *n* = 10; ^***^p < 0.001 *vs*. vehicle-treated control group. ^###^*p* < 0.001 *vs*. vehicle-treated Aβ group. ^$$$^*p* < 0.001 *vs*. rolipram (1.0 mg/kg)-treated Aβ group.

Rolipram-induced memory enhancement was supported by the step-down passive avoidance test, as shown in Figure 4. Treatment of Aβ1-42 induced a significant decrease in step-down latency (the latency to jump off the platform) both 3 and 24 h after training session (*p's* < 0.001, Figures 4A,B). Treatment of rolipram (0.1, 0.5, and 1.0 mg/kg, i.p.) for 14 days significantly enhanced the memory retention [*F*_(3, 36)_ = 20.86, *p* < 0.001, Figure 4A; *F*_(3, 36)_ = 22.93, *p* < 0.001, Figure 4B]. However, PKA inhibitor H89 reversed the effects of rolipram on step-down latency significantly (1.0 mg/kg for 14 days) [*F*_(2, 27)_ = 41.70, *p* < 0.001, Figure 4A; *F*_(2, 27)_ = 34.60, *p* < 0.001, Figure 4B], whereas PKG inhibitor did not show such effects. Neither H89 nor KT5823 used alone showed any effects in MWM and PA in mice treated with Aβ1-42 (Supplementary Figures 1A–G).

### Rolipram reduced the serum corticosterone (CORT) levels, but did not change the ratio of adrenal gland to body weight (AG/B)

**Figure 5:**
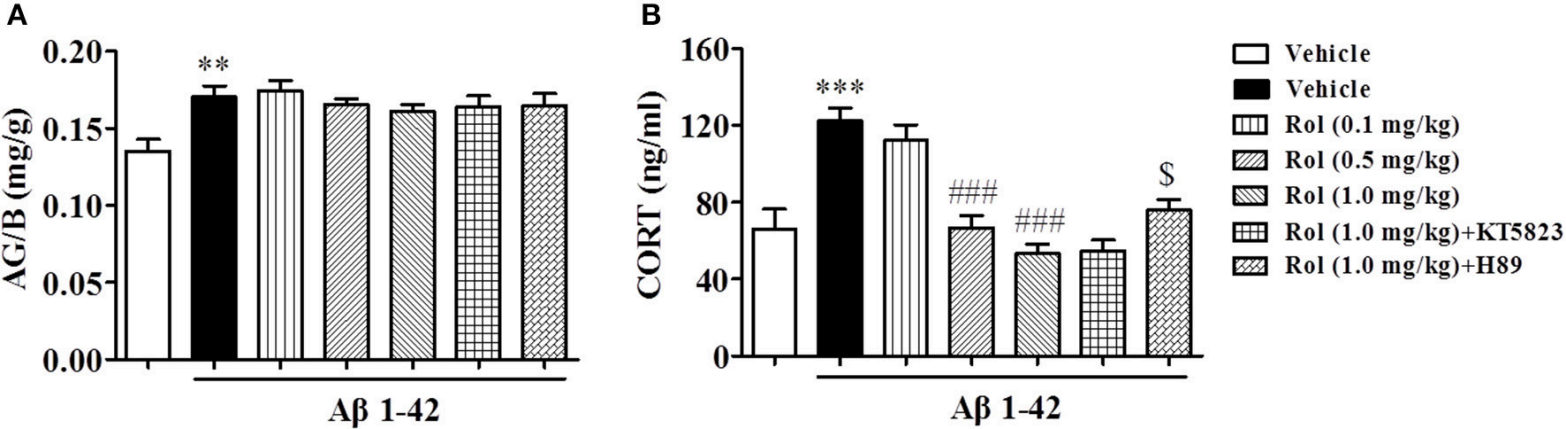
The effects of rolipram on the ratio of adrenal gland weight to body weight (AG/B) (mg/g) **(A)** and serum corticosterone level **(B)** in Aβ42-treated mice. Values shown are means ± SEM, *n* = 10; ^**^p < 0.01 and ^***^p < 0.001 *vs*. vehicle-treated control group. ^###^*p* < 0.001 *vs*. vehicle-treated Aβ group. ^$^*p* < 0.05 *vs*. rolipram (1.0 mg/kg)-treated Aβ group.

The effects of chronic administration with rolipram on the AG/B and serum corticosterone levels in Aβ1-42-treated mice were summarized in Figure 5. Aβ1-42 administration significantly increased the ratio of AG/B relative to vehicle-treated controls (*p* < 0.01). Rolipram (0.1, 0.5, and 1.0 mg/kg, i.p.) did not induce any changes in this ratio [*F*_(3, 36)_ = 0.961, *p* > 0.05, Figure 4A]. However, Aβ1-42 microinjection resulted in a significant elevation of serum CORT level (*P* < 0.001, Figure 5B). Rolipram (0.1, 0.5, and 1.0 mg/kg) significantly reduced the Aβ1-42-induced increase in CORT level compared to vehicle-treated Aβ1-42 group [*F*_(3, 36)_ = 26.43, *p's* < 0.001]. However, pretreatment with H89 partially prevented the reduction of serum CORT level induced by high dose of rolipram at 1.0 mg/kg (*p* < 0.05), whereas KT5823 did not have such positive effects. H89 and KT5823 used alone did not show any specific effects on CORT level (Supplementary Figure 1H).

### Rolipram reversed the Aβ-induced increases in CRF receptor and GR expression in the hippocampus and cortex

**Figure 6:**
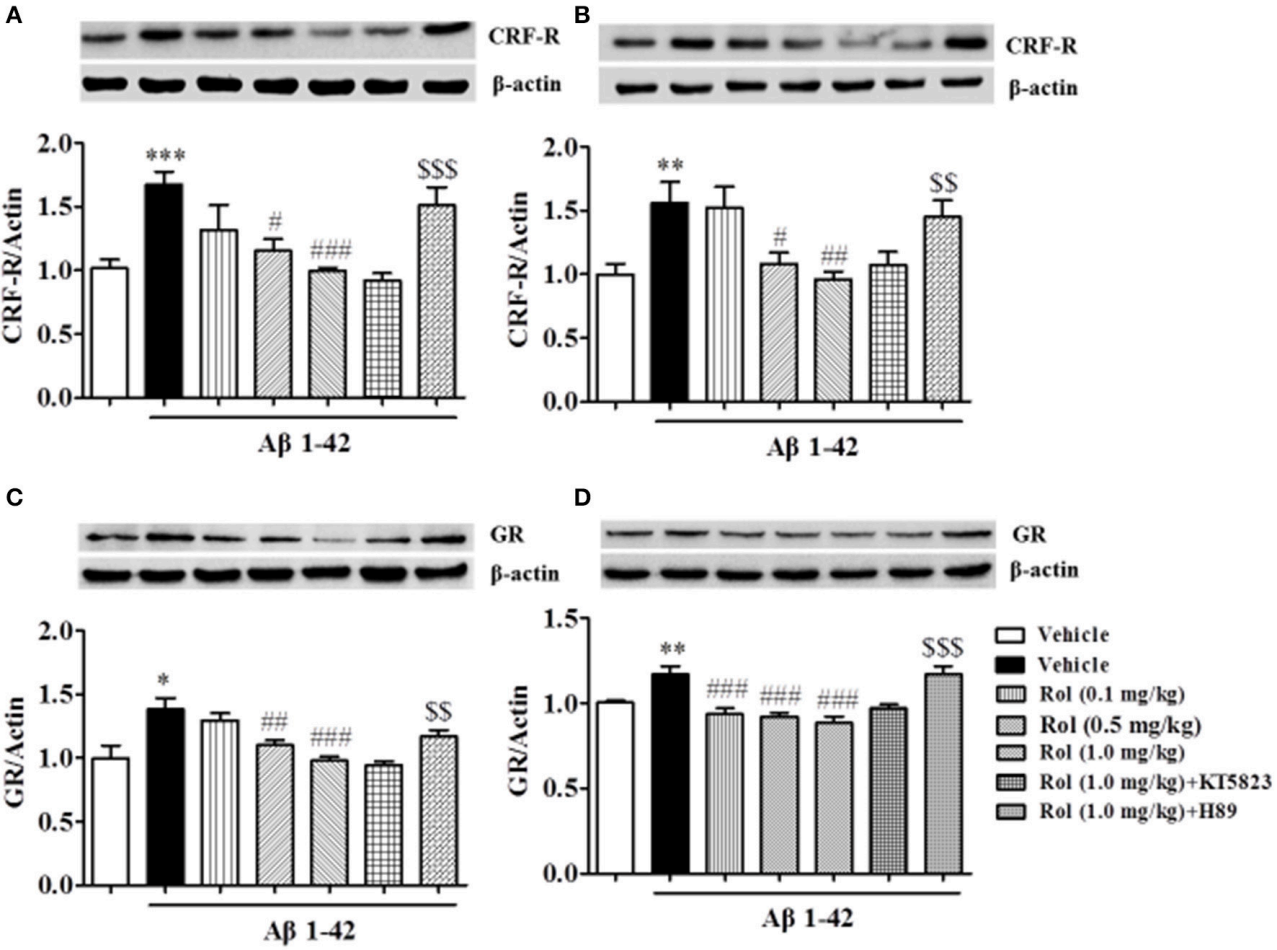
The effects of rolipram on Aβ-induced changes in CRF receptor and GR expression in the hippocampus **(A,C)** and in the cortex **(B,D)** of mice. Values shown are means ± SEM, *n* = 10; ^*^p < 0.05, ^**^p < 0.01, and ^***^p < 0.001 *vs*. vehicle-treated control group. ^#^*p* < 0.05, ^##^*p* < 0.01 and ^###^*p* < 0.001 *vs*. vehicle-treated Aβ group. ^$$^*p* < 0.01 and ^$$$^*p* < 0.001 *vs*. rolipram (1.0 mg/kg)-treated Aβ group.

The expression of CRF receptor in Aβ-treated mice increased in the hippocampus and cortex regions when compared with the respective control groups (*p* < 0.001, *p* < 0.01, Figures 6A,B). Treatment with rolipram (0.1, 0.5, and 1.0 mg/kg, i.p.) for 2 weeks significantly decreased CRF receptor expression both in the hippocampus [*F*_(3, 36)_ = 5.64, *p* < 0.01] and cortex [*F*_(3, 36)_ = 5.59, *p* < 0.01] regions, which were blocked by pretreatment of H89 (*p* < 0.001; *p* < 0.01), but not KT5823. Similarly, GR levels increased in the hippocampus and cortex in Aβ-treated mice (*p* < 0.05; *p* < 0.01, Figures 6C,D). This Aβ1-42-induced increase in GR was significantly blocked by chronic treatment with rolipram at doses of 0.1, 0.5, and 1.0 mg/kg in the hippocampus [*F*_(3, 36)_ = 9.85, *p* < 0.01] and cortex [*F*_(3, 36)_ = 13.85, *p* < 0.001]. However, H89 inhibited the rolipram's effects on GR expression in these two brain regions (*p* < 0.01; *p* < 0.001). Both H89 and KT5823 used alone did not show any effects on CRF receptor and GR expression (Supplementary Figures 2A–D).

### Rolipram reversed the Aβ-induced decreases in pCREB/CREB and BDNF expression in the hippocampus and cortex

**Figure 7:**
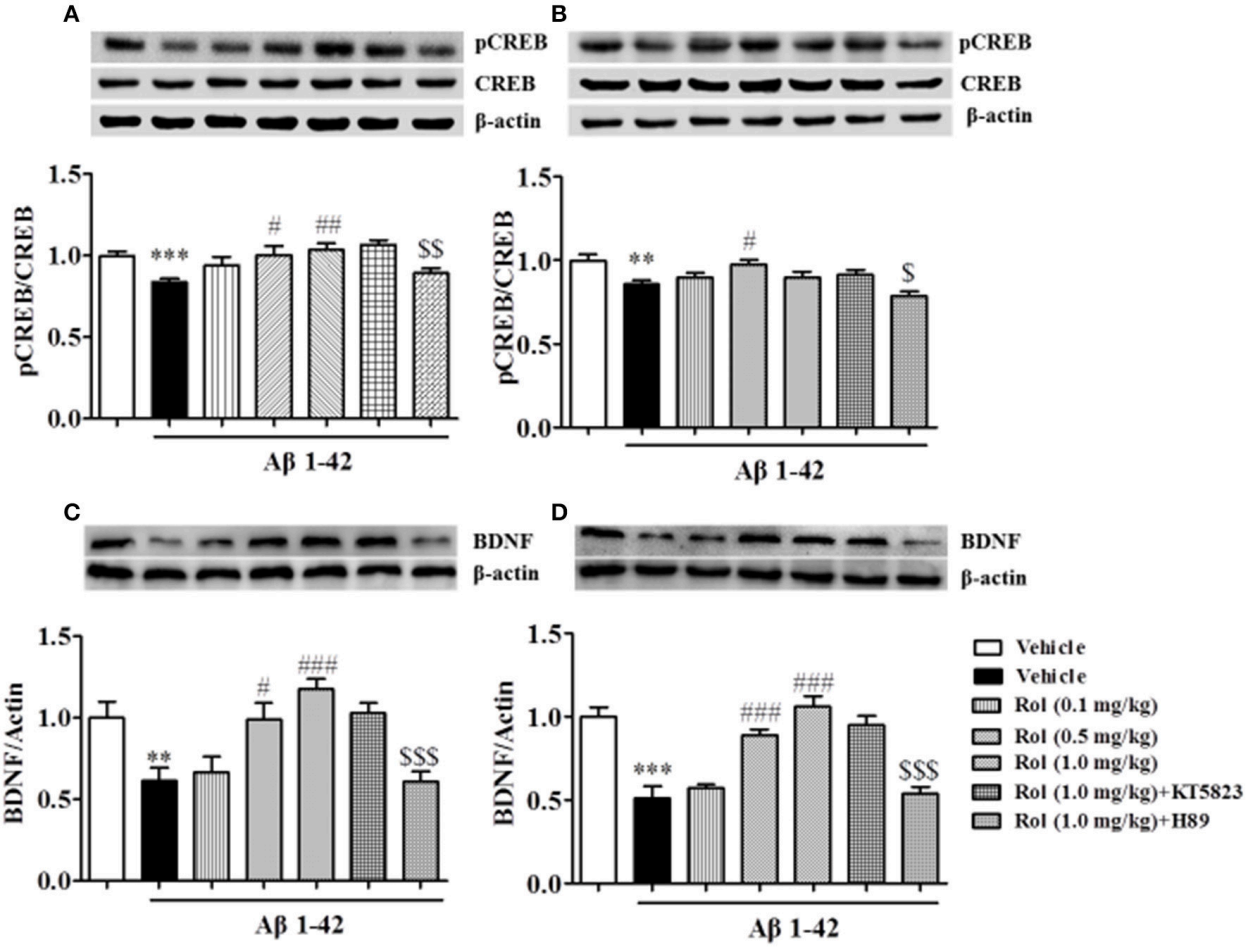
The effects of rolipram on Aβ-induced changes in the ratio of pCREB to CREB and BDNF expression in the hippocampus **(A,C)** and the cortex **(B,D)** of mice. Values shown are means ± SEM, *n* = 10; ^**^p < 0.01 and ^***^p < 0.001 *vs*. vehicle-treated control group. ^#^*p* < 0.05, ^##^*p* < 0.01 and ^###^*p* < 0.001 *vs*. vehicle-treated Aβ group. ^$^*p* < 0.05, ^$$^*p* < ,0.01 and ^$$$^*p* < 0.001 *vs*. rolipram (0.5 or 1.0 mg/kg)-treated Aβ group.

As shown in Figures 7A,B, the reduction of pCREB/CREB caused by the treatment of Aβ1-42 for 2 months was found (*p* < 0.001 in the hippocampus, *p* < 0.01 in the cortex). This reduction was ameliorated by the administration of rolipram for 14 days [*F*_(3, 36)_ = 3.754, *p* < 0.05, Figure 7A; *F*_(3, 36)_ = 3.512, *p* < 0.05, Figure 7B]. The PKA inhibitor H89 reversed the 0.5 or 1.0 mg/kg dose of rolipram-induced increase in the ratio of pCREB to CREB both in the hippocampus and cortex regions (*p* < 0.01; *p* < 0.05).

The significant decreases in BDNF levels in both the hippocampus and cortex regions were observed after treatment with Aβ1-42 (*p* < 0.01; *p* < 0.001; Figures 7C,D). Rolipram at 0.5 and 1.0 mg/kg (i.p.) significantly increased the BDNF levels in both hippocampus (*p* < 0.05, *p* < 0.001) and cortex (*p's* < 0.001). H89 reversed the effects of rolipram on BDNF expression in both of the hippocampus and cortex regions (*p* < 0.001; *p* < 0.001). Both H89 and KT5823 used alone did not show any influence on pCREB/CREB and BDNF expressions (Supplementary Figures 2E–H).

**Figure 8:**
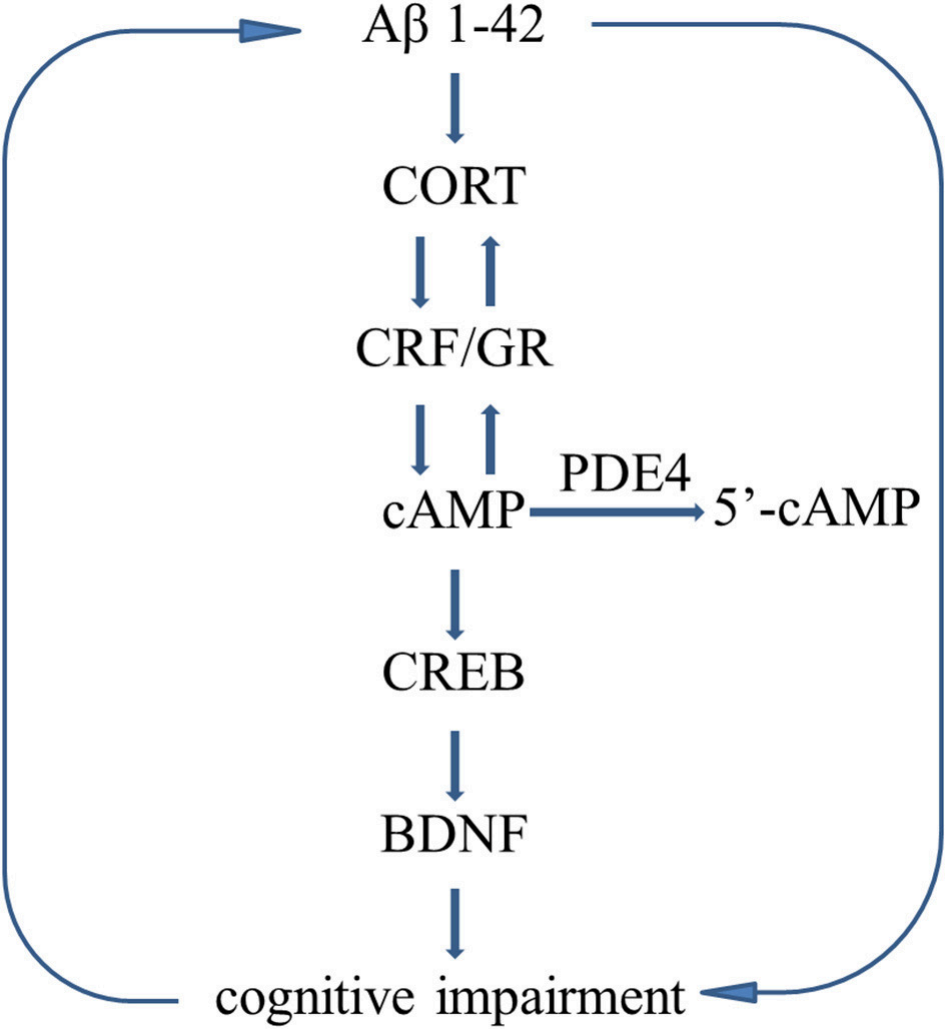
Molecular mechanisms describe the Aβ-induced vicious circle. Aβ1-42 induces HPA axis hyperactivity and decreases the cAMP level, leading to abnormalities in downstream molecules, i.e., decreased phosphorylation of CREB and BDNF expression, which in turn to deteriorate learning and memory disorders. However, Inhibition of PDE4 increases cAMP level and stimulates HPA axis negative feedback regulation, resulting in decreases in CORT releasing and CRF receptor and GR expression.

Figure 8 summarizes the effects of rolipram on Aβ1-42-induced learning and memory impairments and the related signaling pathway.

## Discussion

The present study suggested that Aβ1-42 intracerebroventricular administration caused overall impairment of learning and memory processes in both of the MWM and PA tests at 1 week, 2 and 6 months after treatment, of which the effect was most prominent at 2 months. The subsequent studies demonstrated that the PDE4 inhibitor rolipram was able to ameliorate Aβ1-42-induced learning and memory impairment by regulating the HPA axis, leading to increases in cAMP-related proteins such as the ratio of pCREB/CREB and BDNF expression. However, these effects were blocked by the PKA inhibitor H89, but not the PKG inhibitor KT5823. These findings suggested that rolipram ameliorated the memory impairment through the regulation of Aβ-induced HPA axis dysfunction and initiated the downstream cAMP-dependent neuroprotective pathway.

Aβ deposition in the brain regions such as hippocampus and frontal cortex is considered as an early event in the process of AD, which involves deterioration in learning and memory processes (Wang et al., 2016). A large number of studies have shown that microinfusion of Aβ, particularly Aβ1-42, into the cerebral ventricle is a reliable model of AD because the cerebral ventricle is a communicating network of cavities filled with cerebrospinal fluid (CSF) that transmits messages to other brain regions such as hippocampus and cortex that regulate learning and memory processes (Guo et al., 2012; Thorajak et al., 2017). The Aβ-induced AD animal model mimics the memory impairment and a variety of other pathological features in AD patients such as amyloid plaques and neurofibrillary tangles (Tiraboschi et al., 2004). In the present study, the Morris water maze and step down passive avoidance tests were used to test learning and memory performance at 1 week, 2 and 6 months after intracerebroventricular microinjection of Aβ1-42. The results demonstrated that the animals started to show reduced learning and memory performance in both tests 1 week after Aβ administration; the effect peaked at 2 months after treatment and lasted for at least 6 months. Two reasons may explain this phenomenon: (1) Aβ1-42 induced neurotoxicity would disappear as time goes on; or (2) the adaptive response to Aβ would be stimulated after 6 months. Accordingly, it is reliable that the subsequent cognitive behaviors after treatment with rolipram were conducted at 2 months after microinfusion of Aβ1-42 into cerebral ventricle.

Our previous studies suggested that microinjection of Aβ into the CA1 induced decreased second messengers (cAMP and cGMP) in the hippocampus (Wang L. et al., 2017). As the enzymes that hydrolyze and inactivate the second messenger cAMP, PDE4 is found to be associated with memory regulation. For example, PDE4 inhibitor rolipram is shown to promote memory formation by increasing the concentration of cAMP in the brain (Villiger and Dunn, 1981). Aβ-induced learning and memory impairment and pathological changes including deficits in cAMP signaling are consistent with the performance of early stage of AD patients (Matsuzaki et al., 2006). Our present study suggested that treatment with rolipram (0.1, 0.5, 1.0 mg/kg-d, i.p.) for 2 weeks was able to protect animals against Aβ-induced MWM deficits, as mice received rolipram learned faster in the training session, and rolipram treatment restored memory performance to that of vehicle-treated Aβ mice during the probe trials. These results were confirmed by PA test, which suggested that short-term and long-term memory retention was ameliorated by treatment with rolipram. However, pretreatment of animals with the PKA inhibitor H89, but not the PKG inhibitor KT5823, prevented rolipram-induced protective effects, which supported the notion that the effects of rolipram on enhancing learning and memory were achieved through regulation of cAMP signaling. These were consistent with an earlier finding that the PDE4 inhibitor rolipram selectively inhibited cAMP hydrolysis and upregulated PKA levels (Wang et al., 2014). It was noted in the present study that rolipram started to treat 1.5 months after Aβ1-42 microinjection, in which memory impairment or dementia was getting worse in animals. Moreover, rolipram was shown to improve both short-term and long-term memory, which is different from the past studies in which rolipram primarily enhanced long-term memory (Wang et al., 2012). It is widely accepted that activating the cAMP-dependent pathway might ameliorate hippocampus-dependent long-term memory consolidation (Richter et al., 2013). Indeed, short-term memory is primarily dependent on regions of the prefrontal cortex, whereas the consolidation of information from short-term memory to long-term memory depends on the function of hippocampus (Cohen, 2011; Serences, 2016). In the present study, Aβ1-42 was microinjected into the cerebral ventricle, which permitted Aβ1-42 to transmit messages to various brain regions including hippocampus and cortex that make it possible to impair both short-term and long-term memory processes. Inhibition of PDE4 by intraperitoneal injection might be able to upregulate cAMP signaling in the cortex and hippocampus, which is considered to have impact on both short-term and long-term memory. Moreover, the fact that rolipram regulates the HPA axis dysfunction is also important to enhance short-term memory (Anderson et al., 2014). Therefore, the present results support the hypothesis that inhibition of PDE4 reversed the memory impairment induced by Aβ1-42.

The neuroendocrine disorders such as abnormal glucocorticoids (GCs) levels in AD patients have been reported in previous studies (Landfield et al., 2007; Brureau et al., 2013). The up-regulation of GCs in AD animal model potentially leads to HPA axis dysfunction and aggravates the AD process. Indeed, dysfunction of HPA axis such as abnormalities in CORT secretion has been linked to the changes in CNS disorders, particularly learning and memory impairments (Dong et al., 2008). Previous *in vitro* studies suggested that corticotrophin (ACTH) from pituitary cells appears to be regulated by a cAMP/PKA-mediated pathway (Hadley et al., 1996; Vargas et al., 2001). In the present study, we found that excessive CORT releasing stimulated by Aβ1-42 was rescued by treatment of rolipram for 2 weeks, which was consistent with the previous studies that suggested rolipram inhibited the GR function, whereas PKA activator 8-Br-cAMP increased the GR transcription and function (Dong et al., 1989; Carvalho et al., 2010). Interestingly, H89 significantly blocked the rolipram's effects on CORT releasing, which supports that the effects of rolipram on regulating the HPA axis is mediated through the cAMP-PKA-dependent mechanism (Jindal et al., 2015).

CRF is widely expressed in brain regions and in the types of neuronal populations that are involved in the regulation of cognition, emotion and endocrine function (Regev and Baram, 2014). Increased CRF and corticosterone, in turn, stimulates more Aβ releasing from neurons, which induces deleterious effects on the structure and function of various brain structures such as cortex and hippocampus, leading to deterioration of learning and memory as seen in both animal models and patients with AD (Guo et al., 2012). Corticosterone and CRF easily cross the blood-brain barrier and then bind to their receptors in the brain. The CRF receptor and GR spread all over the brain regions, particularly the hippocampus and the prefrontal cortex, which are involved in negative feedback response of HPA axis. The present study found that rolipram negatively regulated CRF receptor and GR abnormalities in the hippocampus and cortex caused by intraventricular administration of Aβ1-42. Subsequent studies suggested that PKA inhibitor blocked the effects of rolipram on GR and CRF receptor expression, supporting the hypothesis that rolipram could regulate HPA axis through cAMP signaling.

CREB is being considered as a possible therapeutic target for AD. The aggregation of Aβ suppressed the phosphorylation of CREB-mediated signaling in mouse model of AD (Xu et al., 2006), thereby affecting the transcription of genes related to neuronal plasticity and survival, such as BDNF (Pugazhenthi et al., 2011; Xu et al., 2013). Down-regulation of BDNF mediates the toxic effects of prolonged GR activation on neuronal survival and disrupts the normal expression of CRF receptor (Jeanneteau and Chao, 2013; Wang R. et al., 2017), which deteriorate Aβ toxicity. The pathological basis of AD offers a therapeutic opportunity by enhancing the cAMP-related signaling, leading to correction of negative feedback of HPA axis (Zhang et al., 2015). In the present study, Aβ-induced decreases in the ratio of pCREB/CREB and BDNF expression both in the hippocampus and cortex, which were rescued by PDE4 inhibitor rolipram. However, these effects were blocked by H89, but not KT5823, further supporting that cAMP-pCREB-BDNF pathway is involved in rolipram's effects on Aβ-induced memory deficits. The proposed molecular mechanism was summarized in Figure 8.

Taken together, the present study suggested that learning and memory impairments were found from 1 week and it became worst at 2 months after microinjection of Aβ1-42 to the cerebral ventricle. Treatment with the PDE4 inhibitor rolipram for 2 weeks significantly ameliorated the learning and memory deficits. These effects of rolipram might involve the regulation of HPA axis and the downstream cAMP-pCREB-BDNF signaling.

## Author contributions

YX, NZ, and WX wrote the manuscript and did a part of the behavioral tests. HY, KL, FW, MZ, and YD performed the biochemical and neurobiological experiments. CZ analyzed the data. JO and HZ revised the manuscript. JP designed and supervised the manuscript.

### Conflict of interest statement

The authors declare that the research was conducted in the absence of any commercial or financial relationships that could be construed as a potential conflict of interest.

## Supplementary material

Supplementary Figures 1 and 2 The effects of H89 and KT5823 used alone on Aβ-induced cognitive impairment.
