# Supplementary figures and images for: Inhibition of Phosphodiesterase-4 Reverses Aβ-Induced Memory Impairment by Regulation of HPA Axis Related cAMP Signaling
Source: Front Aging Neurosci. 2018 Jul 24;10:204. doi: 10.3389/fnagi.2018.00204 (PMC6066959; doi:10.3389/fnagi.2018.00204)

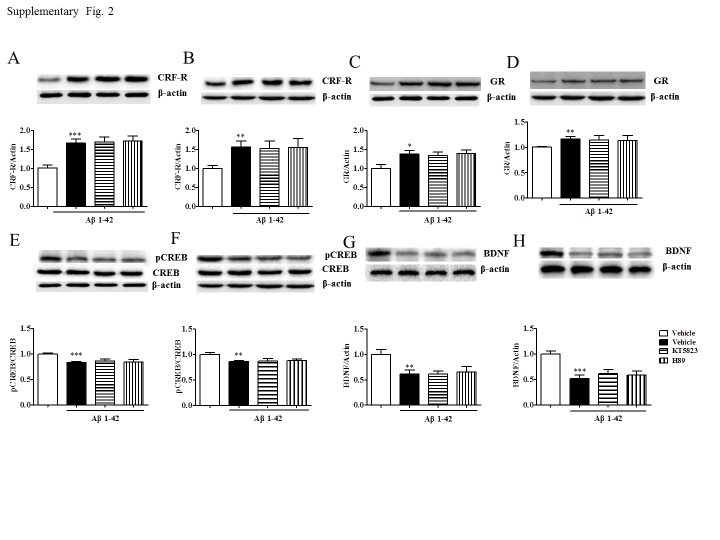

Supplement: Supplementary Figures 1 and 2 — The effects of H89 and KT5823 used alone on Aβ-induced cognitive impairment. [file Image_2.TIF]
